# Methanol leaf extract of *Momordica charantia* protects alloxan-induced hepatopathy through modulation of caspase-9 and interleukin-1β signaling pathways in rats

**DOI:** 10.14202/vetworld.2020.1528-1535

**Published:** 2020-08-08

**Authors:** Sunday Oluwaseun Ofuegbe, Ademola Adetokunbo Oyagbemi, Temidayo Olutayo Omobowale, Aduragbenro Deborah Adedapo, Abiodun Emmanuel Ayodele, Momoh Audu Yakubu, Oluwafemi O. Oguntibeju, Adeolu Alex Adedapo

**Affiliations:** 1 Department of Veterinary Pharmacology and Toxicology, Faculty of Veterinary Medicine, University of Ibadan, Nigeria; 2 Department of Veterinary Physiology and Biochemistry, Faculty of Veterinary Medicine, University of Ibadan, Nigeria; 3 Department of Veterinary Medicine, Faculty of Veterinary Medicine, University of Ibadan, Nigeria; 4 Department of Pharmacology, College of Medicine, University of Ibadan, Nigeria; 5 Department of Botany, Faculty of Science, University of Ibadan, Nigeria; 6 Department of Environmental and Interdisciplinary Sciences, College of Science, Engineering and Technology, NSB303, Vascular Biology Unit, Center for Cardiovascular Diseases, COPHS, Texas Southern University, Houston, TX, USA; 7 Oxidative Stress Research Centre, Phytomedicine and Phytochemistry, Department of Biomedical Sciences, Faculty of Health and Wellness Sciences, Cape Peninsula University of Technology, Bellville 7535, South Africa

**Keywords:** antioxidant, caspase-9, hepatotoxicity, histopathology, interleukin-1β, *Momordica charantia*

## Abstract

**Background and Aim:**

*Momordica charantia* is a highly valued plant, widely distributed in the tropical and subtropical regions. The plant is reported to have a wide range of medicinal uses. This study was designed to explore the ameliorative potential of *M. charantia* methanol leaf extract in alloxan-induced diabetic animal model with a particular focus on the liver.

**Materials and Methods:**

Hepatoprotective effect of methanol leaf extract of *M. charantia* was assessed in alloxan-induced toxicity in 50 rats divided into five groups (A-E) (n=10). Group A normal control, Group B was toxicant group, and Group C animals received glibenclamide treatment while Groups D and E received extracts at 200 and 400 mg/kg doses, respectively. The experiment lasted for 28 days. Histopathological changes, blood glucose level, and serum enzymes such as aspartate aminotransferase, alanine aminotransferase, and alkaline phosphatase, oxidative status and caspase-9, and interleukin-1β (IL-1β) were evaluated.

**Results:**

Extract-treatment caused a decreased blood glucose level, markers of oxidative stress such as malondialdehyde and hydrogen peroxide (H_2_O_2_). Treatment of rats with leaf extract of *M. charantia* resulted in increased levels and activities of protein thiols, non-protein thiols, glutathione (GSH), glutathione peroxidase, glutathione S-transferase, and superoxide dismutase indicating its antioxidant potential. The liver section revealed mild distortion of the hepatic architecture compared to the toxicant group, while decreased expressions of caspase-9 and IL-1β in extract-treated groups was observed.

**Conclusion:**

The plant extract exhibited antioxidant, anti-apoptotic, and anti-inflammatory effects, thus showing its hepatoprotective property.

## Introduction

Liver is the second largest organ in the body involved in various steps of metabolic and physiologic homeostasis, making the liver an important organ. [1]. The liver is essential for detoxification and removal of toxic materials. Diabetogenic agents, including alloxan are known to induce hepatocellular injury [2], eliciting the production of free radicals, leading to oxidative stress, and subsequent depletion of vital antioxidants such as reduced glutathione (GSH). Treatment of liver disorders is inadequate despite the strides being made in medicine; thus, herbal extracts are being explored in the protection against liver damage and hepatic cell regeneration [3]. Persistent hyperglycemia and oxidative stress are major factors leading to diabetic complications including hepatic injury [4]. There are reports that the liver can be greatly compromised by diabetes in the long-term [5].

Plants rich in triterpenes, flavonoids, or polyphenol are known to possess antihepatotoxic activities in experimental liver-injury models [6]. Studies have shown that natural antioxidants found in plants offer protection to the liver against chemical-induced peroxidative damage [7]. *Momordica charantia* L (bitter melon or bitter gourd) of the family Cucurbitaceae is a tropical plant that is widely cultivated in Asia, India, East Africa, and South America and is regarded as a natural therapy for treating diabetes [8]. Its pharmacological actions have been attributed to its high antioxidant content due in part to phenols, flavonoids, isoflavones, terpenes, anthraquinones, and glucosinolates [9]. Many clinical studies have reported that extracts from the fruit, seeds, and leaves of bitter melon have various phytochemicals that have hypoglycemic activity in diabetic conditions [10,11].

This study was designed to explore the ameliorative potential of *M. charantia* methanol leaf extract in alloxan-induced diabetic animal model with a particular focus on the liver.

## Materials and Methods

### Ethical approval

The study protocol was approved by the Animal Care and Use Research Ethics Committee, University of Ibadan, (UI-ACUREC/App/2015/044).

### Study period and location

The experiment was performed in January 2018 at the Department of Veterinary Pharmacology and Toxicology, Faculty of Veterinary Medicine, University of Ibadan, Oyo State, Nigeria.

### Extract preparation

The fresh whole plant of *M. charantia* was collected from the Botanical Garden, University of Ibadan. The identification and authentication of the plant were performed at the Department of Botany, University of Ibadan with Voucher Specimen Number (UIH-22563). The voucher specimen was maintained at the Herbarium of the Department of Botany, University of Ibadan. The leaves were dried at room temperature (27±2°C) and pulverized to a fine powder using an electric blender. The powder (400 g) was soaked and extracted in 90% methanol (1L) using Soxhlet extractor for 3 days until complete extraction. The filtrate was filtered using Whatman no 1 filter paper and the filtrate was evaporated to dryness by a rotary evaporator (Yamato, Rotary Evaporator, model-RE 801, Japan) at 190-220 rpm and 40-50°C for 24 h under reduced pressure to give amorphous solid mass [12]. The extract yield was 12%.

### Animal care and feeding

Fifty healthy white adult male Wistar strain albino rats divided into five Groups A-E (n=10, 150-200 g) obtained from the animal house of the Faculty of Veterinary Medicine, University of Ibadan, Ibadan, were used in this study. The animals were maintained at the Experimental Animal House of the Faculty of Veterinary Medicine, University of Ibadan, in rat cages. They were acclimatized for 2 weeks at room temperature (22±2°C) under a 12 h light/12 h dark cycle with humidity of 55±5%. The animals were allowed free access to clean fresh water and standard rat pellets. The animals received humane care according to the criteria outlined in the guide for the care and use of laboratory animals. All experimental protocols were in compliance with the National Institute of Health Guide for Care and Use of Laboratory animals [13] and the internationally accepted principles for laboratory animal use and care.

### Experimental procedure

Liver toxicity was induced in rats by the administration of freshly prepared alloxan (100 mg/kg) dissolved in distilled water intraperitoneally to induce diabetes [14]. Two days later, blood was drawn from each rat and glucose level measured to establish diabetes. Animals with blood glucose levels>225 mg/dl were considered to be diabetic [15]. Fasting blood glucose (FBG) levels were estimated by commercially available glucose kit glucometer (Roche, Mannheim, Germany). Forty rats with blood glucose >225 mg/dl were considered to be diabetic. The 40 diabetic animals were used in the study in addition to ten normal rats that were not injected with alloxan. The rats were thereafter divided into five groups of ten animals each and the procedure was conducted as follows:

Group A: Normal non-diabetic non-treated control rats received 2.5% Tween 80 in normal saline (NDNT).

Group B: Diabetic control rats received no treatment following the induction of diabetes (DNT) with alloxan.

Group C: Diabetic rats were treated with glibenclamide at 4 mg/kg body weight by daily oral gavage (DTG).

Group D: Diabetic rats received methanol leaf extract of *M. charantia* (MLEMC) at a dose of 200 mg/kg body weight by DTG (DTMC200).

Group E: Diabetic rats received MLEMC at a dose of 400 mg/kg body weight by DTG (DTMC400).

The experiment lasted for 28 days. Evaluation of blood glucose status was performed on days 14 and 28. The assessment of liver damage in alloxan-induced diabetic rats and ameliorative activity of the extract were performed by measuring the activities of serum liver enzymes: Aspartate aminotransferase (AST); alanine aminotransferase (ALT), and alkaline phosphatase (ALP), and oxidative stress markers. Lipid peroxidation assay, antioxidant status, histological examination of liver sections, and immunohistochemical staining of the liver were also carried out.

### Ameliorative effects of the methanol leaf extract of M. charantia (MLEMC)

#### Preparation of serum and tissues for biochemical assays

Blood samples were collected on days 14 and 28 through the retro-orbital plexus using clean heparinized capillary tubes and dry clean plain tubes and allowed to coagulate. The blood samples were then centrifuged at 4000 revolutions/min (rpm) for 15 min. The serum was collected and stored in the refrigerator at −4°C until the time of analysis. The rats were humanely sacrificed on the 29^th^ day after the termination of the experiment by cervical dislocation. The livers were quickly excised, rinsed in cold NDNT, and immediately kept in ice to prevent denaturation of biomolecules [16].

### Biochemical assays

Hydrogen peroxide was determined, as described by Wolff [17]. Lipid peroxidation (malondialdehyde, [MDA] level) was determined by measuring the formation of thiobarbituric acid reactive substances according to the methods of Varshney and Kale [18]. Renal non-protein thiol (NPSH) and protein thiol (PSH) content were determined by the method of Sedlak and Lindsay [19]. Renal GSH (reduced glutathione) level was estimated by the method of Jollow *et al*. [20]. The renal glutathione peroxidase (GPx) activity was measured according to Rotruck *et al*. [21]. Glutathione S-transferase (GST) activity was determined according to Habig *et al*. [22]. The activity of superoxide dismutase (SOD) in the renal homogenates was determined by the method of Misra and Fridovich [23]. Serum urea and creatinine level were determined using Randox kits according to manufacturer’s instructions.

### Immunohistochemistry of hepatic caspase-9 and interleukin 1-Beta (IL-1β)

Immunohistochemistry was done from paraffin sectioned hepatic tissues, as described by Oyagbemi *et al*. [24].

#### Histopathological studies

Examination of liver histology was performed according to routine histology techniques, as described by Drury *et al*. [25].

### Statistical analysis

Results were expressed as Mean ± standard deviation (SD). Statistical analysis was performed using the statistical package GraphPad prism version 5 (GraphPad software, San Diego CA, USA). The significant difference in the means was determined using one-way analysis of variance (ANOVA; 95% confidence interval). Tukey *post hoc* tests were performed for comparison of all groups with control and comparison of all pairs of groups, respectively. Values of α_0.05_ were considered as significant [26].

## Results

### Effects of MLEMC on blood glucose and serum liver enzymes of alloxan-induced diabetic rats

The effects of MLEMC on liver functions and blood glucose levels are shown in Table-1. The serum levels of hepato-specific enzymes (AST, ALT, and ALP) were significantly elevated in the diabetic rats due to hepatocellular injury. The rats treated with MLEMC and glibenclamide showed significant reduction in the elevated activities of liver enzymes. In the case of blood glucose level, results showed that at day 28, all the groups treated with glibenclamide and MLEMC experienced significant decrease in their values.

**Table-1:** Effects of the methanol leaf extract of *Momordica charantia* on serum liver enzymes of alloxan-induced diabetic rats.

Parameter	A(NDNT)	B(DNT)	C(DTG)	D(DTMC200)	E(DTMC400)
AST(U/L)					
Basal	41.40±4.34	41.14±1.95	41.43±4.12	40.88±3.56	41.57±1.62
Day 14	42.33±2.79	48.75±3.10^αγ^	41.80±2.49^ε^	43.00±2.51^ε^	44.75±2.66^αε^
Day 28	44.00±7.31	50.67±5.86^αγ^	43.00±2.94^ε^	40.60±2.61^ε^	42.60±1.94^ε^
ALT(U/L)					
Basal	29.60±2.97	29.86±1.68	29.00±2.71	29.13±2.75	30.29±2.75
Day 14	30.78±3.45	42.75±3.50^αγ^	34.60±4.51^αγε^	33.63±3.42^αγ^	31.38±3.02^εδ^
Day 28	30.20±2.17	42.67±1.15^αγ^	32.50±3.51^αγε^	30.20±2.86^βεσ^	31.80±2.17^ε^
ALP(U/L)					
Basal	90.20±3.35	91.86±7.58	90.43±6.67	91.63±4.63	91.47±5.88
Day 14	91.14±8.91	109.75±8.42^αγ^	97.80±13.35^αγε^	90.63±8.99^εσ^	92.38±7.96^εδ^
Day 28	91.20±8.53	115.67±3.41^αβδ^	90.50±5.44^βε^	90.20±1.19^ε^	92.80±8.96^ε^
Glu(mg/dl)					
Basal	74.00±3.36	72.60±3.79	81.50±3.34	72.20±3.06	72.90±2.44
Day 14	88.80±3.00	294.37±3.53^a^	184.75±2.26^abc^	242.13±2.64^abc^	177.70±3.83^abc^
Day 28	90.60±3.86	293.00±4.52^a^	136.25±3.69^abcde^	142.00±3.23^abcde^	141.20±3.84^abcde^

Values expressed as mean±SD, n=10. ^αβγεδ^indicate statistical significance (α_0.05_) when compared with basal values, at day 14, normal control, diabetic control, and standard drug respectively. ^abcde^indicate statistical significance (α_0.05_) in glucose level when compared with basal values, at day 14, normal control, diabetic control, and standard drug respectively. Group A NDNT (non-diabetic non-treated), Group B DNT (diabetic non-treated), Group C (diabetic treated with glibenclamide), Group D (diabetic treated with 200mg/kg MC), Group E (diabetic treated 400mg/kg MC). AST=Aspartate aminotransferase, ALT=Alanine aminotransferase, ALP=Alkaline phosphatase, Glu=Blood glucose level

### Effects of MLEMC on the hepatic oxidative damages

In this study, administration of alloxan induced oxidative stress by significantly (α_0.05_) increasing hydrogen peroxide (H_2_O_2_) and MDA levels in the liver post-mitochondrial fractions of the diabetic non-treated rats compared with the normal control. Concurrent administration of MLEMC (200 mg/kg and 400 mg/kg) and glibenclamide (4 mg/kg) significantly (α_0.05_) reduced the level of these oxidative stress markers (H_2_O_2_ and MDA) when compared with the diabetic non-treated rats. Administration of both doses of the extract reduced the hepatic hydrogen peroxide and MDA levels to near normal levels (Table-2).

**Table-2:** Effects of the methanol leaf extract of * Momordica charantia* on markers of oxidative stress in the hepatic tissues of alloxan-induced diabetic rats.

Parameter	Group A	Group B	Group C	Group D	Group E
H_2_O_2_	36.67±1.23	40.21±1.14^α^	34.45±2.16^β^	35.74±2.08^β^	34.76±2.68^β^
MDA	1.31±0.17	1.68±0.22^α^	1.38±0.55^β^	1.36±0.18^β^	1.33±0.28^β^

Results expressed in Mean±SD, n=10. ^α^Significant increase when compared with normal control, ^β^Significant reduction when compared with diabetic control at α_0.05_. H_2_O_2_: Hydrogen peroxide (µmole/min/mg protein); MDA=Malondialdehyde, (μmol formed MDA/mg protein). Group A (normal non treated control), Group B (alloxan-induced non treated), Group C (alloxan-induced treated with glibenclamide), Group D (alloxan-induced treated with 200mg/kg MC), Group E (alloxan-induced treated with 400mg/kg MC)

### Effects of the MLEMC on the antioxidant system of the hepatic tissues of alloxan-induced diabetic rats

In this study, diabetic rats showed a reduction in the level of PSH and NPSH in the hepatic post-mitochondrial fractions (PMF) compared with the control (Table-3). The level of protein and NPSH thiols significantly (α_0.05_) increased in the hepatic PMF of MLEMC treated groups compared with the diabetic rats. Furthermore, administration of alloxan resulted in significant (α_0.05_) decrease in the content of reduced glutathione (GSH) in the hepatic post-mitochondrial fractions of the diabetic rats compared with the control group (Table-3). Treatment with MLEMC and glibenclamide induced significant (α_0.05_) increase in the content of GSH compared with the diabetic group (Table-3).

**Table-3:** Effects of the methanol leaf extract of *Momordica charantia* on the antioxidant system of the hepatic tissues of alloxan-induced diabetic rats.

Parameter	Group A	Group B	Group C	Group D	Group E
Non-protein thiol	18.00±2.38	14.66±1.20	16.83±2.07	19.00±1.13^β^	18.71±2.50^β^
Protein thiol	47.12±1.50	42.23±1.50^α^	47.27±1.90^β^	46.79±2.30^β^	47.32±1.98^β^
Glutathione	95.13±3.71	90.51±2.65^α^	94.04±2.59^β^	94.26±2.32^β^	94.59±2.00^β^

Results expressed in Mean±SD, n=10. ^α^Significant compared with diabetic control at α_0.05_^β^Significant compared with diabetic control at α_0.05_. NPSH and PSH=Non protein thiol and protein thiol, respectively (μmol /mg protein), GSH=Reduced glutathione (μmol /mg protein). Group A NDNT=Non-diabetic non-treated, Group B DNT=Diabetic non-treated, Group C (diabetic treated with glibenclamide), Group D (diabetic treated with 200 mg/kg MC), Group E (diabetic treated 400 mg/kg MC)

### Effects of the MLEMC on antioxidant defense system components (GPx, GST, and SOD) of hepatic tissues of alloxan-induced diabetic rats

Significant (α_0.05_) decrease was observed in the activities of GPx, glutathione S-transferase (GST), and SOD in the hepatic post-mitochondrial fractions of the diabetic rats compared with the control group. There was significant (α_0.05_) increase in the activities of antioxidant enzymes in the MLEMC and glibenclamide treated rats compared with the diabetic non-treated group (Table-4).

**Table-4:** Effects of the methanol leaf extract of *Momordica charantia* on Antioxidant enzymes of hepatic tissues of alloxan-elicited diabetic rats.

Parameter	Group A	Group B	Group C	Group D	Group E
GPx	32.31±1.86	21.57±1.85^α^	32.48±1.17^β^	33.51±1.95^β^	33.87±1.41^β^
GST	0.31±0.09	0.22±0.05^α^	0.30±0.02^β^	0.28±0.09^β^	0.30±0.05^β^
SOD	11.33±1.43	9.57±1.20^α^	94.04±2.59^β^	12.22±0.77^β^	11.78±0.54^β^

Results expressed in Mean±SD, n=10. ^α^Significant reduction when compared with normal control, ^β^Significant increase when compared with diabetic control at α_0.05_. GPx=Glutathione peroxidase, (µmol/mg protein), GST=Glutathione S-transferase (mmol 1-chloro-2,4-dinitrobenzene-GSH complex formed/min/mg protein), SOD=Superoxide dismutase, units/mg protein. Group A NDNT=Non-diabetic non-treated, Group B DNT=Diabetic non-treated, Group C (diabetic treated with glibenclamide), Group D (diabetic treated with 200 mg/kg MC), Group E (diabetic treated 400 mg/kg MC)

### Effects of the MLEMC on the antibodies expression in the hepatic tissues of alloxan-induced diabetic rats

**Figure-1:**
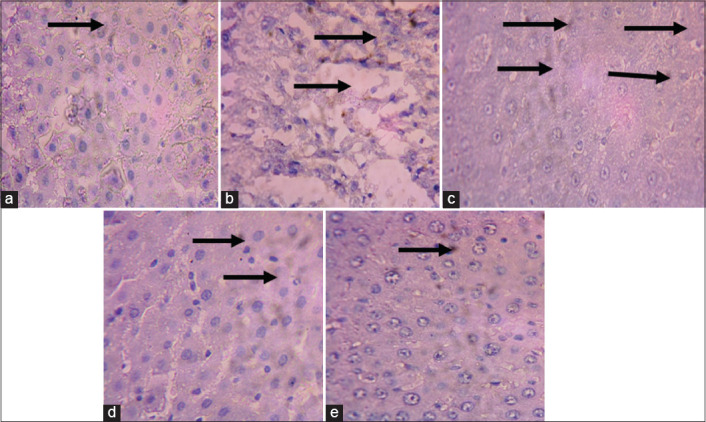
Immunohistochemical staining of liver caspase-9. (a) control (non-diabetic non-treated) shows low expression of caspase-9, (b) diabetic non-treated shows higher expression of caspase-9 than control, (c) daily oral gavage shows lower expression of caspase-9 similar to control, (d) DTMC200 shows lower expression of caspase-9 similar to control, (e) DTMC400 shows lower expression of caspase-9 similar to control. The slides were counterstained with high definition hematoxylin and viewed 400× objectives.

**Figure-2:**
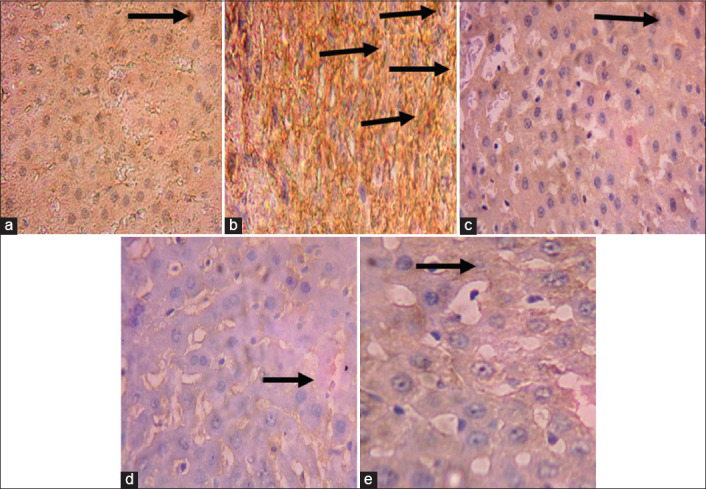
Immunohistochemical staining of liver interleukin-1β (IL-1β). (a) Control (non-diabetic non-treated) shows low expression of IL-1β, (b) diabetic non-treated shows a higher expression of IL-1β than control, (c) daily oral gavage shows lower expression of IL-1β similar to control, (d) DTMC200 shows the lower expression of IL-1β similar to control, (e) DTMC400 shows the lower expression of IL-1β similar to control. The slides were counterstained with high definition hematoxylin and viewed 400×.

There was a higher expression of caspase-9 (Figure-1b) and IL-1β (Figure-2b) in the liver of the diabetic rats when compared with the control rats (Figures-1a and 2a). There was low expression of these proteins in the MLEMC and glibenclamide treated rats (Figures-1c-e and 2c-e) similar to the control rats.

### Effects of the MLEMC on the hepatic tissues of alloxan-induced diabetic rats

**Figure-3:**
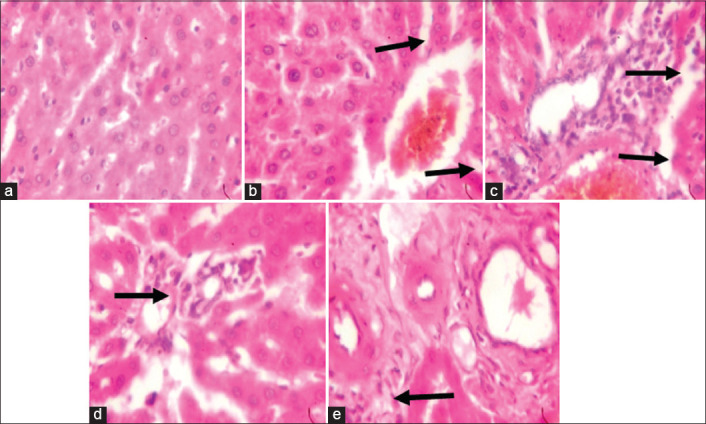
Photomicrograph of liver. (a) (non-diabetic non-treated) No visible lesion, (b) diabetic non-treated showed congestion and necrosis, (c) daily oral gavage showed infiltration of inflammatory cells and congestion. (d) DTMC200 showed moderate infiltration of inflammatory cells while (e) DTMC400 showed moderate infiltration of inflammatory cells (400×, H and E).

The result of histological changes induced in the liver by alloxan administration and treatment with MLEMC and glibenclamide is presented in Figures-3a-e. Histological examination of tissue sections of the liver revealed that the control group (Figure-3a) showed normal histoarchitecture of the liver parenchyma with characteristic appearance of hepatic lobule. The hepatocytes are seen radiating from the central vein separated by sinusoids and portal tract/triad (hepatic portal vein, hepatic vein, and bile duct). The diabetic non-treated group (Figure-3b) showed severe distortion of the histoarchitecture of the liver with congested central vein; necrosis; sinusoidal dilatation; and vacuolation. The liver section of the glibenclamide treated group (Figure-3c) showed mild distortion of the histoarchitecture of the liver, area of congestion, and infiltration by inflammatory cells and necrosis. The liver section of the MLEMC treated groups (Figure-3d and e) revealed mild distortion of the hepatic architecture showing that the extract reduced the histopathological abnormalities that were seen in the liver of diabetic rats. Liver sections from rats treated with MLEMC showed recovery of the hepatocytes from necrosis and other pathological changes indicating that the extract preserved the structural integrity of the hepatocellular membrane and liver cell architecture.

## Discussion

Diabetes mellitus is characterized by progressive metabolic derangement, hyperglycemia, and morphological changes in the liver, kidney, retina, pancreas, and other organs [27]. In all these abnormalities, oxidative stress is a major risk factor [2]. Hepatic injury as a result of hyperglycemia leads to enlargement of sinusoids, dilation of cisterns, as well as a loss of rough endoplasmic reticulum ribosomes in the liver cells [28]. Therefore, the changes in the activities of serum liver enzymes (AST, ALT, and ALP) as well as morphological and histopathological lesions found in the livers of the animals in this study are attributed to diabetes-induced chronic stress.

Increased activities of these enzymes as seen in alloxan-induced diabetic-untreated rats in this study corresponds to the extensive liver damage induced by the toxicant (alloxan). In this study, the elevated serum activities of hepatic enzymes (ALT, AST, and ALP) were significantly reduced by MLEMC treatment. This may be due to stabilization of plasma membrane as well as repair of damaged hepatic tissue [29]. The restoration of these serum liver enzymes compared to their normal activities after treatment with MLEMC shows revival of the liver and regeneration of the hepatocytes by the extract. This supports the ameliorative potential of the plant.

Furthermore, the results of this study showed a significant increase in lipid peroxidation and elevated level of hydrogen peroxide content in the liver of alloxan-induced diabetic rats, indicating oxidative stress. This confirms the report on the ability of diabetogenic compounds to induce oxidative damage through the generation of free radicals [30]. However, the administration of MLEMC reduced hepatic MDA and hydrogen peroxide levels, thereby substantiating its antioxidant ability.

There was a marked decrease in the content of hepatic non-enzymic antioxidants (PSH, NPSH, and GSH) and activities of enzymic antioxidant defense system (GPx, GST, and SOD) in alloxan-induced diabetic untreated rats. This is an indication of oxidative stress induced by alloxan. However, MLEMC administration significantly increased the levels and activities of these antioxidant systems (both enzymic and non-enzymic), indicating the antioxidant activities of this plant. Plants rich in triterpenes, flavonoids, or polyphenol are known to possess antihepatotoxic activities in the experimental liver-injury models [6]. Studies have shown that the natural antioxidants found in plants offer protection to the liver against chemical-induced peroxidative damage [6]. The plant, *M. charantia* is said to have many medicinal properties such as hepatoprotective, antidiabetes, anti-oxidative, anti-inflammatory, hypoglycemic, antibacterial, antiviral, antitumor, anthelmintic, and antifertility activities [31]. In this study, the ability of the plant extract to increase the antioxidant enzymes confirms this. Furthermore, the plant extract significantly reduced the blood glucose level on day 28, indicating its antidiabetic and hypoglycemic properties. The hepatoprotective property of this plant is also demonstrated in this study due to its ability to significantly reduce the activities of liver enzymes to near normal. The plant is able to demonstrate all these activities due to its rich bioactive phytochemicals such as polysaccharides, saponins, and phenolics [32,33], hence its antioxidant activity against oxidant damage *in vitro* and *in vivo* [34,35] models.

Immunohistochemical staining of the liver revealed a high expressions of caspase-9 and IL-1β in the alloxan-induced diabetic rats. However, there was reduced expression of these proteins in the liver of MLEMC treated rats. Caspase-9 is an initiator caspase [36], which is activated during programmed cell death (apoptosis). Downregulation of caspase-9 expressions by MLEMC may imply that the extract protects the hepatic tissue against TNF-receptor-activated apoptosis. This may establish the anti-apoptotic potential of MLEMC. IL-1 beta is a potent pro-inflammatory cytokine, which induces prostaglandin synthesis, neutrophil influx, and activation. It has been shown that increase in the level of IL-1β is a risk factor for the development of type 2 diabetes [37]. The downregulation of IL-1β by MLEMC in the liver implies its anti-inflammatory ability.

Histologically, significant hepatic injury characterized by hypertrophic and necrotic hepatocytes, collapse of the central veins, widespread inflammatory cells infiltration round the central vein, and loss of cellular boundaries were observed in the untreated diabetic group. Our finding agrees with the work of Bilal *et al*. [38]. However, in rats supplemented with MLEMC (200 and 400 mg/kg), the extent of hepatic lesions was ameliorated relative to the lesions seen in untreated rats. The administration of MLEMC reduced liver pathological conditions, suggesting that the plant has the ability to enhanced regeneration.

The amelioration can also be ascribed to the occurrence of flavonoids, terpenes, and tannins [39]. These constituents possess free-radical scavenging, anti-oxidative, and anti-lipoperoxidive properties, which promotes hepatoprotection. *M. charantia* also contains proanthocyanidins, known to inhibit peroxidative liver injury. Furthermore, Ning *et al*. [40], Li *et al*. [41] and Shehab *et al*. [42] reported that plants containing triterpenoids and flavonoids are useful in treating liver disorders. Consequently, the hepatoprotective action of MLEMC could be credited partly to its inherent phytochemicals. The outcomes of this work validated the ethnomedicinal value of *M. charantia* in the management of hepatitis.

This study has shown that extracts of MLEMC at 200 and 400 mg/kg displayed tangible mark of amelioration and notable recovery of the architecture of the liver in alloxan-induced hepatic injury.

## Conclusion

It can be concluded from this study that *M. charantia* has a potential to ameliorate biochemical and histological changes in the liver of alloxan-induced diabetic Wistar rats. The amelioration of liver damage exhibited by the MLEMC was comparable to glibenclamide, the standard drug used in this study. In addition, this study revealed that *M. charantia* possesses anti-apoptotic and anti-inflammatory potentials.

## Authors’ Contributions

SOO, AAO, TOO, and ADA carried out the experiment, AAA, MAY, and AEA designed and wrote the draft of the manuscript. OOO edited the manuscript. All authors read and approved the final draft of the manuscript.

## Competing Interests

The authors declare that they have no competing interests.

## Publisher’s Note

Veterinary World remains neutral with regard to jurisdictional claims in published institutional affiliation.

## References

[1] Chiang J, Linda M Mcmanus, Richard N, Mitchell (2014). Liver Physiology: Metabolism and Detoxification. Pathobiology of Human Disease.

[2] Amanda N.L, Lucas L.C, César T.S. (2015). Alloxan-induced diabetes causes morphological and ultrastructural changes in rat liver that resemble the natural history of chronic fatty liver disease in humans. J. Diabetes Res.

[3] Hong M, Li S, Tan H.Y, Wang N, Tsao S, Feng Y. (2015). Current status of herbal medicines in chronic liver disease therapy:The biological effects, molecular targets and future prospects. Int. J. Mol. Sci.

[4] Mohamed J, Nafizah A.H.N, Zariyantey A.H, Budin S.B. (2016). Mechanisms of diabetes-induced liver damage:The role of oxidative stress and inflammation. Sultan. Qaboos. Univ. Med. J.

[5] Mendes-Braz M, Martins J.O. (2018). Diabetes mellitus and liver surgery: The effect of diabetes on oxidative stress and inflammation. Med. Inflamm.

[6] Adeyemi O.D, Ukwenya O.V, Obuotor E.M, Adewole O.S. (2014). Anti-hepatotoxic activities of *Hibiscus sabdariffa* L. in animal model of streptozotocin diabetes-induced liver damage. BMC Complement. Altern. Med.

[7] Sha L, Hor-Yue T, Ning W, Zhang-Jin Z, Lixing L, Chi-Woon W, Yibin F. (2015). The role of oxidative stress and antioxidants in liver diseases. Int. J. Mol. Sci.

[8] Bhat G.A, Khan H.A, Alhomida A.S, Sharma P, Singh R, Paray B.A. (2018). GLP-I secretion in healthy and diabetic Wistar rats in response to aqueous extract of *Momordica charantia BMC Complement*. Altern. Med.

[9] Rasheedat B, Akinwande A.I, Magbagbeola O.A, Wahab O (2010). Nutritional and chemical evaluation of *Momordica charantia*. J. Med. Plant Res.

[10] Hazarika R, Parida P, Neog B, Yadav R.N.S. (2012). Binding energy calculation of GSK-3 protein of human against some anti-diabetic compounds of *Momordica charantia* Linn (Bitter melon). Bioinformation.

[11] Wehash F.E, Abpo-Ghanema I.I, Saleh R.M. (2012). Some physiological effects of *Momordica charantia* and *Trigonella foenum-graecum* extracts in diabetic rats as compared with Cidophage®. World Acad. Sci. Eng. Technol.

[12] Omale J, Enemuor S.C, Hussaaini E.E. (2010). Antimicrobial and antioxidant activity of *Saba florida* (Benth) extracts. J. Biosci. Biotech.

[13] National Institute of Health (1985). Guide for the Care and Use of Laboratory Animals.

[14] Patel N, Raval S, Goriya H, Jhala M, Joshi B. (2007). Evaluation of antidiabetic activity of *Coldenia procumbens* in alloxan-induced diabetes in rat. J. Herb. Pharmacother.

[15] Thirumalai T, Viviyan T.S, Elumalai E.K, David E. (2011). Hypoglycemic effect of *Brassica juncea* (seeds) on streptozotocin-induced diabetic male albino rat. Asian Pac. J. Trop. Biomed.

[16] Nagy M.A (2015). Antioxidant and antiapoptotic effects of *Cystoseira myrica* on hepatic dysfunction in alloxan-induced diabetes mellitus in male albino rats. BCAIJ.

[17] Wolff S.F (1994). Ferrous ion oxidation in the presence of ferric ion indicator xylenol orange for measurement of hydrogen peroxides. Methods Enzymol.

[18] Varshney R, Kale R.K. (1990). Effect of calmodulin antagonists on radiation induced lipid peroxidation in microsomes. Int. J. Radiat. Biol.

[19] Sedlak J, Lindsay R.H. (1968). Estimation of total protein-bound and nonprotein sulfhydryl groups in tissue with Ellman's reagent. Anal. Biochem.

[20] Jollow D.J, Mitchell J.R, Zampaglione N, Gillette J.R. (1974). Bromobenzene-induced liver necrosis: Protective role of glutathione and evidence for 3,4-bromobenzene oxide as the hepatotoxic metabolite. Pharmacology.

[21] Rotruck J.T, Pope A.L, Ganther H.E, Swanson A.B, Hafeman D.G, Hoekstra W.G (1973). Selenium: Biochemical role as a component of glutathione peroxidase *Science*.

[22] Habig W.H, Pabst M.J, Jakoby W.B. (1974). Glutathione-S-transferases. The first enzymatic step in mercapturic acid formation. J. Biol. Chem.

[23] Misra H.P, Friedovich I. (1972). The role of superoxide anion in the autoxidation of epinephrine and a simple assay for superoxide dismutase. J. Biol. Chem.

[24] Oyagbemi A.A, Omobowale T.O, Akinrinde A.S, Saba A.B, Ogunpolu B.S, Daramola O. (2014). Lack of reversal of oxidative damage in renal tissues of lead acetate-treated rats. Environ. Toxicol.

[25] Drury R.A, Wallington E.A, Cancerson R. (1976). Carlton's Histopathological Techniques.

[26] Betty R.K, Jonathan A.C. (2003). Essential Medical Statistics.

[27] Verderese J.P, Younossi Z. (2013). Interaction of Type 2 diabetes and nonalcoholic fatty liver disease. Expert Rev. Gastroenterol. Hepatol.

[28] Lucchesi A.N, de Freitas N.T, Cassettari L.L, Marques S.F.G, Spadella C.T. (2013). Diabetes mellitus triggers oxidative stress in the liver of alloxan-treated rats:A mechanism for diabetic chronic liver disease. Acta. Cir. Bras.

[29] Fang G, Zhen-Hua Y, Qitai X, Wen-Yi K. (2012). Hepatoprotective effect of *Mitragyna rotundifolia* Kuntze on CCl4-induced acute liver injury in mice. Afr. J. Pharm. Pharmacol.

[30] Tangvarasittichai S (2015). Oxidative stress, insulin resistance, dyslipidemia and Type 2 diabetes mellitus. World J. Diabetes.

[31] Ahmed I, Adeghate E, Sharma A.K, Pallot D.J, Singh J. (1998). Effects of *Momordica charantia* fruit juice on islet morphology in the pancreas of the streptozotocin-diabetic rat. Diabetes Res. Clin. Pract.

[32] Raza H, Ahmed I, John A, Sharma A.K. (2000). Modulation of xenobiotic metabolism and oxidative stress in chronic streptozotocin-induced diabetic rats fed with *Momordica charantia* fruit extract. J. Biochem. Mol. Toxicol.

[33] Virdi J, Sivakami S, Shahani S, Suthar A.C, Banavalikar M.M, Biyani M.K. (2003). Antihyperglycemic effects of three extracts from *Momordica Charantia*. J. Ethnopharmacol.

[34] Bajpai M, Pande A, Tewari S.K, Prakash D. (2005). Phenolic contents and antioxidant activity of some food and medicinal plants. Int. J. Food Sci. Nutr.

[35] Liu C.H, Yen M.H, Tsang S.F, Gan K.H, Hsu H.Y, Lin C.N. (2010). Antioxidant triterpenoids from the stems of *Momordica charantia*. Food Chem.

[36] Bratton S, Salvesen G. (2010). Regulation of the Apaf-1-caspase-9 apoptosome. J. Cell Sci.

[37] Maedler K, Dharmadhikari G, Schumann D.M, Storling J (2009). Interleukin-1? targeted therapy for Type 2 diabetes. Expert Opin. Biol. Ther.

[38] Bilal H.M, Riaz F, Munir K, Saqib A, Sarwar M.R. (2016). Histological changes in the liver of diabetic rats: A review of pathogenesis of nonalcoholic fatty liver disease in Type 1 diabetes mellitus. Cogent. Med.

[39] Rex J.R.S, Muthukumar N.M.S.A, Paulraj M.S (2018). Phytochemicals as a potential source for anti-microbial, antioxidant and wound healing - a review. MOJ Biorg Org Chem.

[40] Ning W, Peibo L, Yonggang W, Wei P, Zhong W, Suiyi T. (2008). Hepatoprotective effect of *Hypericum japonicum* extract and its fractions. J. Ethnopharmacol.

[41] Li S, Tan H, Wang N, Zhang Z, Lao L, Wong C, Feng Y. (2015). The role of oxidative stress and antioxidants in liver diseases. Int. J. Mol. Sci.

[42] Shehab N.G, Abu-Gharbieh E, Bayoumi F.A (2015). Impact of phenolic composition on hepatoprotective and antioxidant effects of four desert medicinal plants. BMC Complement. Altern. Med.

